# Therapeutic applications of adipose cell-free derivatives: a review

**DOI:** 10.1186/s13287-020-01831-3

**Published:** 2020-07-22

**Authors:** Yuan Cai, Jianyi Li, Changsha Jia, Yunfan He, Chengliang Deng

**Affiliations:** 1 Department of Dermatology, Affiliated Hospital of Zunyi Medical University, Zunyi, 563000 Guizhou People’s Republic of China; 2 Department of Plastic Surgery, Affiliated Hospital of Zunyi Medical University, Zunyi, 563000 Guizhou People’s Republic of China; 3 Department of Plastic Surgery, Nanfang Hospital, Southern Medical University, 1838 Guangzhou North Road, Guangzhou, 510515 Guangdong People’s Republic of China

**Keywords:** Adipose-derived stem cell, Cell-free, Conditioned medium, Exosome, Adipose tissue extract

## Abstract

**Background:**

Adipose-derived stem cells (ADSCs) have become one of the most utilized adult stem cells due to their abundance and accessibility. Recent studies have shown that paracrine cytokines, exosomes, and other active substances are the main factors through which ADSCs exert their biological effects.

**Main body:**

Adipose cell-free derivatives have been recently gaining attention as potential therapeutic agents for various human diseases. These derivatives include ADSC-conditioned medium (ADSC-CM), ADSC exosomes (ADSC-Exo), and cell-free adipose tissue extracts (ATEs), all of which can be conveniently carried, stored, and transported. Currently, research on ADSC-conditioned medium (ADSC-CM) and ADSC exosomes (ADSC-Exo) is surging. Moreover, cell-free adipose tissue extracts (ATEs), obtained by purely physical methods, have emerged as the focus of research in recent years.

**Conclusion:**

Adipose cell-free derivatives delivery can promote cell proliferation, migration, and angiogenesis, suppress cell apoptosis, and inflammation, as well as reduce oxidative stress and immune regulation. Thus, adipose cell-free derivatives have a broad therapeutic potential in many areas, as they possess anti-skin aging properties, promote wound healing, reduce scar formation, and provide myocardial protection and neuroprotection. This article summarizes these effects and reviews research progress in the use of adipose cell-free derivatives.

## Background

Adipose tissue consists of a stromal vascular fraction (SVF), mature adipocytes, and an extracellular matrix (ECM). The SVF includes adipose-derived stem cells (ADSCs), adipose precursor cells, endothelial cells, and macrophages [1]. Autologous fat grafting can replenish soft tissues and exert a filling effect; furthermore, native ADSCs present in adipose tissue can play a regenerative role and promote the migration, proliferation, and secretory activity of keratinocytes and fibroblasts [2]. Additionally, adipose tissue modulates the body’s endocrine system by secreting hormones, such as adiponectin and leptin [3]. It was reported that, compared with serum from a lean human group, the fat production trend of MSCs treated with serum of overweight people increased, which may be attributed to less adiponectin and leptin secretion by adipose tissue in obese or overweight people [4, 5]. Therefore, fat grafting is currently widely used in the field of plastic surgery and is gaining increasing attention in the field of regenerative medicine. Recently, adipose tissue extracts (ATEs)—rich in cytokines and extracellular vesicles and obtained by purely physical methods—have emerged as the primary focus of research [6].

In 2005, the International Society of Cell Therapy defined mesenchymal stem cells as class of cells with plasticity, multi-directional differentiation potential and expressing specific surface antigens such as CD73, CD90, and CD105 [7]. These cells have a low ethical burden in application and possess anti-inflammatory, immunomodulatory, and high regeneration characteristics. Therefore, mesenchymal stem cells show great potential in the treatment of many diseases, including immune and non-immune diseases [8]. ADSCs are a type of MSCs derived from the adipose tissues and have the potential for self-renewal and multidirectional differentiation as well as the ability to secrete hundreds of cytokines [9]. Because of the plethora of their sources and abundance, ADSCs are considered one of the most promising adult stem cell types for clinical application and are widely used for tissue engineering and regenerative medicine research [10]. Reportedly, paracrine cytokines, exosomes, and other active substances are the main factors through which ADSCs exert their biological effects [11, 12]. Interestingly, ADSC-conditioned medium (ADSC-CM) and ADSC exosomes (ADSC-Exo) have recently been gaining attention as alternative approaches to conventional ADSC therapy.

**Fig. 1:**
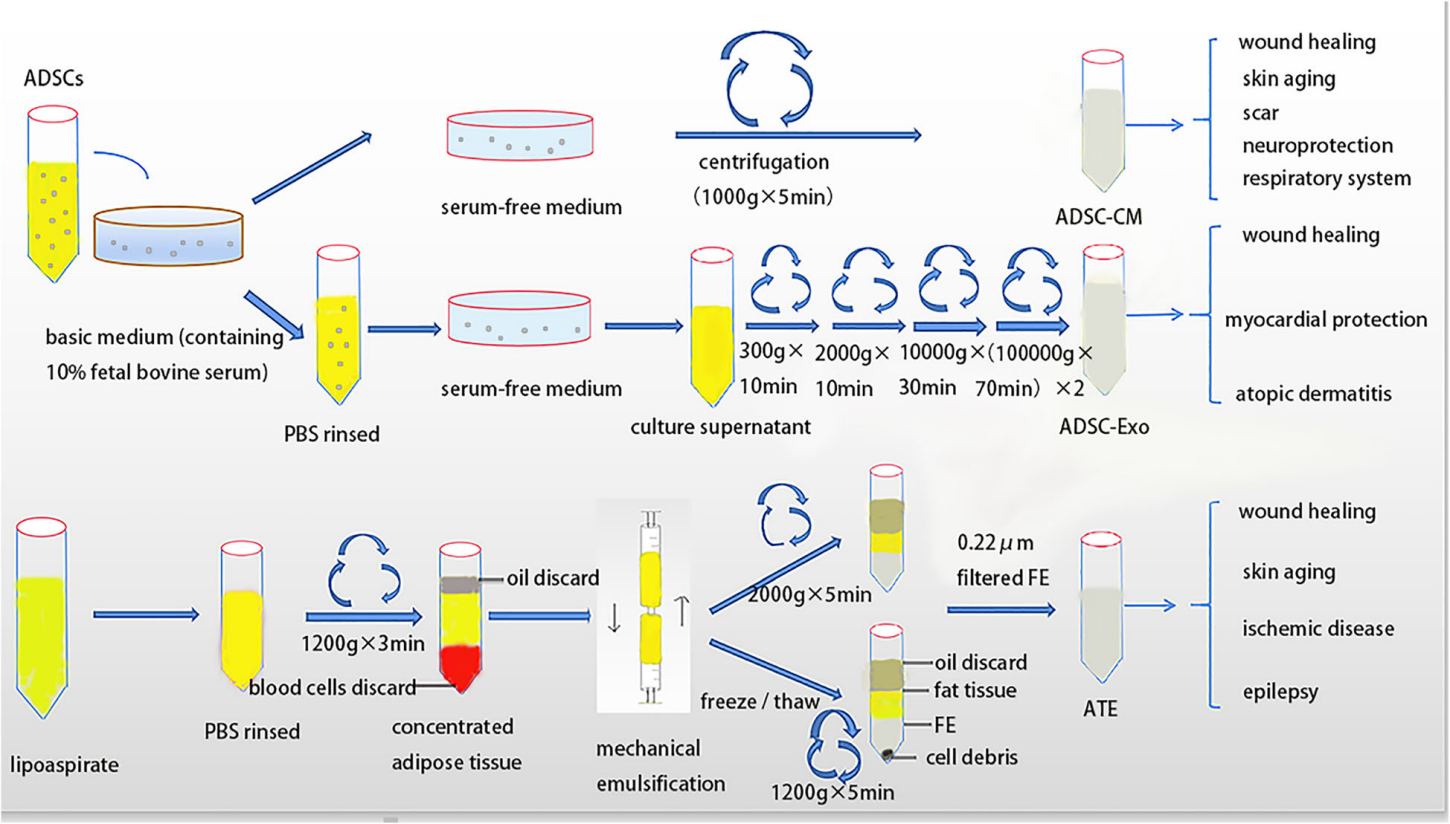
Schematic diagram showing preparation of adipose-derived stem cells conditioned medium, adipose-derived stem cell exosomes, and adipose tissue extracts and its clinical application

Adipose cell-free derivatives include ADSC-CM, ADSC-Exo, and ATEs. ADSC-CM—which contains ADSC-secreted active substances, such as cytokines, exovesicles, exosomes, DNA, and RNA—can promote tissue repair and regulate immunity [13]. ADSC-Exo are extracellular vesicles that carry proteins, RNA, DNA, and lipid molecules; they can penetrate physiological tissue barriers and participate in the exchange of materials and information between cells [14]. As adipose cell-free derivatives do not contain cells and, thus, cannot actively contribute to tumorigenesis, they can be used for allogeneic transplantation. Adipose cell-free derivatives are easy to carry, transport, and store; they have a wide therapeutic potential for the treatment of diseases such as skin aging, wound repair, scar repair, and nerve regeneration. Therefore, this review summarizes the preparation (Fig. 1 and Table 1), storage, and therapeutic uses (Table 2,Table 3,Table 4) of adipose cell-free derivatives.

**Table 1:** Summary of the preparation of adipose cell-free derivatives

Derivative	Preparation	Difference	Potential functional molecule
ADSC-CM	1. Add ADSC to basal medium containing 10% fetal bovine serum for cultivation 2. When ADSC reaches 70–80% confluence, change the basic medium to serum-free medium 3. After cultivating for 48–72 h, collect the medium and centrifuge at 1000 g × 5 min, 4. Filter with a 0.22-μm syringe	Rich in cytokines, but enzyme digestion and in vitro cultivation are required during the preparation process, increasing the risk of biological contamination	bFGF [15, 16]; EGF [15]; PDGF-AA [15, 17]; VEGF [15] KGF [16]; TGF-β [16]; HGF [16, 18], IGF-1 [44]; BDNF [19]
ADSC-Exo	1. Add ADSC to basal medium containing 10% fetal bovine serum for cultivation 2. When ADSC reaches 70–80% confluence, change the basic medium to serum-free medium 3. After 48 h incubation, collect the supernatant and centrifuge 4. To remove any cells and cellular debris, the medium is centrifuged at 300 g for 10 min, 2000 g for 10 min and 10,000 g for 30 min 5. Then, the medium is ultracentrifuged at 100,000 g for 70 min. 6. Finally, Exos at the bottom of the centrifuge tube are resuspended in PBS and centrifuged at 100,000 g for 70 min to eliminate contaminating proteins	In addition to cytokines, it is also rich in signaling molecules such as protein, mRNA, and miRNA, but the preparation steps are complicated, time-consuming, cumbersome to operate, easily mixed with impurities, and have low yield	miR-146a [20]**;** micoRNA-21 [73]**;** MiR-181b-5p [21]**;** GDNF、FGF-1、BDNF、IGF-1、NGF [22]
ATE	1. The lipoaspirate is left in ice water and discard the liquid portion 2. Then, the collected adipose tissue layer is washed with PBS 3. To remove remaining blood cells and other components, the adipose tissue is centrifuged at 1200 g for 3 min 4. The collected adipose tissue is mechanically emulsified 5. Then centrifuge at 2000 g for 5 min and filter with a 0.22-μm syringe filter 6. Or after being frozen and thawed for one cycle and centrifuge at 1200 g for 5 min, then filter with a 0.22-μm syringe filter	Rich in cytokines, but the source is relatively limited	VEGF [23–26] bFGF [24–27] BDNF [23, 27];GDNF [23, 27] TGF-β [23, 26, 27]; HGF [23, 26, 27]; PDGF [23, 26, 27]; IGF-1 [80, 85]; KGF [28]

**Table 2:** Therapeutic uses of ADSC-CM

Ref.	Author	Research in vitro	Research in vivo	Results
[29]	Eun Young Lee	HDFs were incubated with norCM or hypoCM	Two circular full-thickness wounds of 8 mm diameter were created on the backs of the mice.	1. HypoCM promotes HDFs migration and type I collagen secretion and significantly reduced the size of the wound area and depth 2. The wound-healing effect of hypoCM was significantly reduced by the addition of the antibodies of both VEGF and bFGF
[30]	Denise R. Cooper	Co-culture of ADSC-CM with HDF	Rat model of ischemic wound healing	1. ADSC-CM promotes HDF migration and accelerates closure of ischemic wounds. 2. There was no difference between unconditioned media or ADSC-CM for non-ischemic wounds.
[31]	Talita Stessuk	Co-culture of ADSC-CM and PRP with fibroblasts and keratinocytes		1.PRP and ADSC have therapeutic potential for healing and re-epithelialization of chronic wounds.
[15]	Jiajia Zhao	Co-culture of ADSC-CM with HDF		1. EGF, PDGF-AA, VEGF, and bFGF had high concentrations in ADSC-CM. 2. The migration of skin fibroblasts could be significantly stimulated by VEGF, bFGF, and PDGF-AA, and the proliferation could be significantly stimulated by bFGF and EGF in ASC-CM.
[32]	Min Ho Kim	The HaCaT cells and HDF were incubated with the ADSC-CM-2D or ADSC-CM-3D		1. ADSC-CM-3D has a more significant effect on the proliferation and migration of fibroblasts and keratinocytes, the reason may be related to galectin1 expression only in 3D cultured ADSC
[33]	Chengliang Deng	Fibroblast and keratinocyte were cultured in Gel-CM, SVF-CM, or serum-free medium	Full-thickness skin wound of diabetic rats	1. Gel-CM promoted the proliferation and migration of fibroblasts & keratinocytes and increased collagen synthesis in fibroblasts 2. The wound-healing rate in the Gel-CM-treated group was significantly higher than that in the SVF-CM-treated group at all timepoints.
[34]	Chengliang Deng	Fibroblasts and keratinocytes were cultured in Gel-CM, Adi-CM, and SVF-CM or serum-free medium.	BALB/c nude mice wound model	1. Gel-CM-treated group achieved complete wound healing, whereas the other groups still had unhealed wounds 2. Higher expression of bFGF, EGF, and TGF-b in Gel-CM than in other two CMs and a significantly higher expression of VEGF in Gel-CM than in SVF-CM
[35]	Bing-rong Zhou		Twenty-two subjects with Fitzpatrick phototypes III and IV, aged 24 to 50	1. ADSC-CM + FxCR can increase skin elasticity, improve skin surface roughness, and reduce transepidermal water loss, reduce pigmentation after laser 2. ADSC-CM increased dermal collagen density, elastin density, and arranged them in order.
[36]	Xi Wang		30 female volunteers, skin type III and IV, aged 40 to 63	1. Microneedles + ADSC-CM can improve skin roughness, reduce melanin content, increase skin brightness, gloss, elasticity, and anti-wrinkle effects
[16]	Woo-Chan Son	Co-culture of ADSC-CM with UV irradiation of HDF		1. MMP-1 expression was significantly increased in retinoic acid-treated group and both 50 and 100% AdMSC-CM 2. Type 1 procollagen level was significantly increased in TGF-β1-treated group and both 50 and 100% AdMSC-CM treated group
[17]	Shu Guo	Co-culture of different senescent HDF with ADSC-CM before UVA irradiation		1. ADSC-CM pretreatment was significantly reduced HDF aging rate. 2. ADSC-CM up-regulated the expression of type I, type III collagen and elastin, and downregulated the expression of MMP-1 and MMP-9 mRNA
[37]	Lu Li	HaCaTs and NHDFs were irradiated with UV and cultured with 50% and 100%ADSC-CM		1. Both 50% and 100% ADSC-CM treatment can reduce ROS levels 2. ADSC-CM reduces the production of MMP-1 and the secretion of IL-6 by down-regulating UVB-induced MAPK and TGF-β/Smad signaling pathways 3.100% ADSC-CM treatment, the mRNA expression of procollagen type I was gradually increased in HaCaTs and NHDFs
[38]	Xiuxia Wang	Keloid fibroblasts were cultured in ADSC-CM		1. ADSC-CM reduced the ECM-related gene expression in KFs and inhibited cell proliferation and migration 2. ADSC-CM depleted CD31+/CD34+ vessels and reduced collagen deposition
[39]	Qi Zhang		Rabbit ear hypertrophic scar model	1. Both ADSC and ADSCs-CM treatments reduce scar hypertrophy 2. ADSCs were more effective than ADSCs-CM in reducing hypertrophic scars
[40]	Yan Li	HS tissues were cultured with ADSC-CM in the presence of a p38 inhibitor and activator	BALB/c mouse excisional model	1. ADSC-CM decreased the expression of Col1, Col3, and α-SMA in HSFs and suppressed collagen deposition in cultured HS tissues 2. ADSC-CM suppressed scar formation through the inhibition of the p38/MAPK signaling pathway in HSFs in vitro and the anti-fibrosis effect of ADSC-CM was mediated by the p38/MAPK signaling pathway in BALB/c mouse excisional models in vivo
[41]	Junnan Chen	HSFs were cultured in CFSC-CM or control medium		1. CFSC-CM inhibited HSF proliferation and migration 2. CFSC-CM inhibited HSF ECM protein expression
[18]	Ji Ma	HSFs were treated with ADSCs-CM.		1. HGF secreted by ADSC shows anti-fibrotic effect and ADSC-CM attenuates collagen production in HSFs 2. High concentrations of ADSC-CM can inhibit the Col1/Col3 ratio, reduce TIMP-1 levels, and up-regulates MMP-1 expression
[42]	Xing Shan		Adult male rabbit ears acne vulgaris scar model	1. Almost all acne scars were cured after ADSC+CM injection in the rabbit ear acne scar model. 2. ADSC + CM reduces levels of TNF-α, IL-1α, MMP2, and keratin 16
[43]	Peng Hao	Co-culture of ADSC-CM and glutamate-induced neurons		1. ADSC-CM reduced glutamate-induced neuronal injury with a maximum protective effect at 50% CM and neuronal LDH release and trypsin-positive cells 2. ADSC-CM can rescue glutamate-induced neuronal energy depletion
[44]	Yu Jin Cho	HUVECs cultured in 1 ml EGM complete media supplemented ahADSC-CM	Rats MCAO model	1. Continuous infusion of ahADSC-CM can significantly improve functional and structural recovery after stroke 2. Continuous infusion of ahADSC-CM significantly reduced the number of TUNEL-positive cells and Iba1 / TUNEL-positive cells and increased the number of CD31 + microvessels
[19]	Xing Wei		Hypoxic-ischemic brain injury model in neonatal rats	1. AdSC-CM markedly attenuated both short-term and long-term effects of HI-induced brain damage and the deficit in spatial learning and memory associated with HI 2. IGF-1 and BDNF contained in ADSC-CM play an important role in the recovery of neuropathic injury and significantly reduce the long-term functional cognition and motor skills impairment of hypoxic-ischemic brain injury in rats
[45]	Hongyan Lu	HPAEC were treated with ADSC-CM	Mouse lipopolysaccharide-induced ARDS model	1. ADSC-CM-pretreated HPAEC displayed less severe changes in response to H2O2, with attenuated gap formation and ADSC-CM treatment markedly suppressed the LPS-induced protein increase 48 h post-injection. ADSC-CM leads to lung recruitment of neutrophils with a reduced potential for oxidative response. 2. The LPS-induced level of VEGF in BALF was markedly suppressed in ADSC-CM-treated mice
[46]	Anandharajan Rathinasabapathy		Mouse pulmonary hypertension model and pulmonary fibrosis model	1. ADSC-CM treatment arrest the progression of PH by improving ventricular dynamics and attenuating cardiac remodeling and improves the pulmonary vascular remodeling associated with PH 2. ADSC-CM can prevent the progression of PF in the BLEO model in a model of rat PF induced by bleomycin (BLEO)

**Table 3:** Therapeutic uses of ADSC-Exo

Ref.	Author	Research in vitro	Research in vivo	Results
[47]	Zhi Liu	Exosomes labeled with PKH26 were incubated with the MCM cells		1. The PKH26-labeled exosomes were taken up by MCM cells 2. Anti-apoptotic effects of ADSC-exosomes on MCM cells under oxidative stress
[48]	Xiaojun Cui		Rats myocardial I/R and H/R model	1. ADSCs-ex protect against I/R-induced myocardial injury and suppress H/R-induced cell injury in H9c2 cells in vitro 2. ADSCs-ex activate Wnt/b-catenin signaling to protect against myocardial I/R injury
[49]	Huiyu Xu		Male SD rats myocardial infarction model	1. The LVEF and LVFS of rats in the MI + BM-Exo, MI + AD-Exo, and MI + UC-Exo groups were significantly higher than the maternal stem cells 2. The apoptosis of cardiomyocytes and infarction area were significantly reduced in the MI + ADMSC and MI + AD-Exo groups
[50]	Shengqiong Deng		Mice myocardial infarction model	1. ADSC-Exo exert a protective effect on myocardial injury by reversing MI-induced myocardial fibrosis and apoptosis and attenuate MI-induced inflammation by promoting macrophage M2 polarization 2. S1P/SK1/S1PR1 signaling pathway participated in the cardioprotective effects ADSC-Exo
[20]	Junjie Pan	H9c2 cells were transfected with the miR-146a overexpression vector or the EGR1 overexpression vector alone or both in combination	Male Sprague-Dawley (SD) rats myocardial infarction model	1. MiR-146a abundant exosomes are more protective against suppressed AMI-induced myocardial damage 2. The expression of miR-146a in H9c2 inhibited hypoxic-induced myocardial cell injury by suppressing EGR1
[22]	Vesna Bucan	The DRG neurons were cultured with and without exosomes	Adult rats sciatic nerve injury model	1. ADSC-Exo can increase the axon length of dorsal root ganglion (DRG) neurons and regenerate damaged nerve
[51]	Jing Chen	SCs were cultured in the serum-free SCM added with PBS or ASC-Exos	Male Sprague Dawley (SD) rats sciatic nerve injury model	1. ADSC-Exo could be easily internalized by Schwann cells (SCs) and significantly promoted their proliferation and migration 2. ASC-Exos increase neurotrophic factor expression and neurite outgrowth 3. The implantation of ASC-Exos improve sciatic nerve regeneration in vivo
[52]	Nianhua Feng	DiI-labeled ADSC-Exo were cocultured with BV2 cells		1. ADSC-Exo suppressed the activation and decreased the toxicity of LPS-stimulated BV2 cells 2. ADSC-Exo inhibited neuroinflammation by suppressing NF-kB and MAPK signal pathway
[21]	Yujia Yang	BMECs were subjected to OGD for 4 h and then cultured with ADSC-Exo		1. ADSC-Exo contribute to angiogenesis of BMECs following OGD in vitro through microRNA-181b/TRPM7 axis.
[53]	Mijung Lee		Mice in vitro HD model	1. ADSC-Exo treatment can reduce Huntington protein aggregation, improve mitochondrial dysfunction, and reduce the rate of apoptosis
[54]	Mijung Lee	NSCs from G93A ALS mice model used in vitro ALS model		1. ADSC-exo reduces mutant SOD1 aggregation in G93A neuronal cells 2. ADSC-exo reduce abnormally expressed mitochondrial functional proteins, and restore the normal cell phenotype of amyotrophic lateral sclerosis
[55]	Takeshi Katsuda	PKH67-labeled exosomes were incubated with N2a cells		1. ADSC-exo- contain enzymatically active NEP and can be transferred to the neuroblastoma cell line N2a to reduce its intracellular Aβ level and reduce the accumulation of Aβ in the brain
[56]	Li Hu	Skin fibroblasts were co-cultured with ASCs-Exos	Mice skin wound model	1. Internalization of exosomes by fibroblasts and ASCs-Exos promoted fibroblasts migration, proliferation, collagen synthesis in vitro. 2. ASCs-Exos promoted collagen expression and cutanenous wound healing in vivo than local injection group
[57]	Wei Zhang	The HDFs were cocultured with different concentrations of ADSC-Exos	20 male Balb/c mice full-thickness square wound	1. ADSC-Exos activate intracellular collagen secretion in HDFs via the PI3K/Akt signaling pathway and induce the expression of growth factors in vitro 2. ADSC-Exos promote cutaneous wound healing in mice
[58]	Tao Ma	HaCaT cells were cultured with ADSC-Exo		1. ADSC-Exo promote cell proliferation, migration, and inhibit cell apoptosis of HaCaT cells impaired by H_2_O_2_ 2. ADSC-Exo activates Wnt/β-catenin signaling to prompt wound healing
[59]	Chen Yang	The HaCaT cells with or without pretreated with miR-21 plasmid were treated with the ADSCs with or without GW4869 pretreated、AD-exos	Full layer skin wound BALb/c mice model	1. AD-exos and miR-21 could improve the migration and proliferation of the HaCaT cells 2. ADSC-Exos could improve the healing velocity in the full layer wound model of BALb/c mouse and higher miR-21 expression were detected in the experiment groups 3.The excess TGF-βI had negative feedback influence on the miR-21 expression 4. MiR-21 could enhance the MMP-9 and TIMP-2 protein expression but not MMP-2 and TIMP-1 protein via the PI3K/AKT pathway
[60]	K. LIU		Female nude mice acute cutaneous wound healing model	1. The most prominent wound closure was observed in the ASC-Exo + HA group. 2. ASC-Exo + HA could markedly promote fibroblast activities, re-epithelialization and vascularization in wound healing
[61]	Xue Li	The Dil-labeled or denatured exosomes were incubated with EPCs	Adult female Sprague Dawley rats diabetic skin wound model	1. ADSC-Exo reduce glucose-induced EPC senescence 2. Exosomes derived from Nrf2 overexpressing ADSCs inhibit ROS and inflammatory cytokine expression and promote cutaneous wound healing
[62]	Byong Seung Cho		Biostir®-AD cream-induced NC/Nga mice atopic dermatitis model	1. ASC-Exo reduced the serum IgE levels, the number of inflammatory cells such as CD86 + and CD206 + and the symptoms of atopic dermatitis 2. ADSC-Exo can down-regulate the levels of IL-4, IL-23, IL-31, and TNF-α mRNA in a dose-dependent manner.

**Table 4:** Therapeutic uses of ATE

Ref.	Author	Research in vitro	Research in vivo	Results
[24]	Yunfan He	Adipogenesis and angiogenesis induction of ALE	C57BL/6 mice wound model	1. ALE enhanced wound healing in mice 2. ALE induced angiogenesis and adipogenesis in vitro and in vivo
[28]	Jenny F. Lo’pez	ASC, fibroblasts, endothelial cells, and keratinocytes were cultured with ATE		1. ATE contains higher concentrations of KGF and promotes keratinocyte proliferation 2. ATE stimulates fibroblast and adipose stem cell migration
[63]	You Kyung Na	HDF was incubated with ATSC-Ex	Mice circular cutaneous wound model	1. Topical application of ATSC-Ex accelerates wound healing in vivo 2. ATE promotes HDF proliferation and migration and ECM protein production
[64]	Mingwu Deng	Dermal fibroblasts were cultured with different concentrations of FE and then were exposed to UVB light	Female BALB/c nude mice skin photoaging model	1. FE increases cell proliferation and abrogates UVB irradiation-induced cell cycle arrest. 2. FE prevents UVB-Induced cell aging and intracellular ROS, promoted the expression of GPX-1 and COL-1 in vitro. 3. Antiaging effects of FE on the skin of UVB-irradiated nude mice
[26]	Yuda Xu	Fibroblasts were treated with Ceffe	Female BALB/c nude mice skin photoaging model	1. Ceffe increased the proliferation of human skin fibroblasts 2. Ceffe enhances ECM production and inhibits collagen degradation
[23]	Ziyou Yu	The HUVECs were treated with different concentrations of FE	Nude mice hindlimb ischemia model	1. FE attenuated tissue necrosis in a hindlimb ischemia mouse model and enhanced HUVEC proliferation and migration in a dose-dependent manner 2. FE improved HUVEC tube formation in vitro and promoted vascular formation in vivo as detected using the Matrigel assay
[27]	Yizuo Cai	HUVECs were treated with increasing concentrations of FE	Rat skin flap model	1. Fat extract contains multiple growth factors and increases the proliferation, migration, and tube formation of human umbilical vein endothelial cells 2.FE improves skin flap survival in rats and skin flap angiogenesis
[65]	Daejong Jeon		Male C57BL/6J mice epilepsy model	1. ASCs-E does not affect the seizure threshold and severity 2. ASCs-E have an antiepileptogenic effect in the pilocarpine-induced epilepsy model 3. ATE can reduce the number of spontaneous recurrent epilepsy (SRS) and reduce epilepsy behavior in pilocarpine-induced mouse epilepsy

### ADSC-CM

#### Harvesting ADSC-CM

Harvesting ADSC-CM is relatively simple and straightforward (Fig. 1). Generally, basal medium (containing 10% fetal bovine serum) is used for in vitro ADSC culture. At 70–80% confluence, the basal medium is replaced with serum-free medium. After 2–3 days of in vitro culture, the supernatant (ADSC-CM) is collected by centrifugation [66]. The amounts of cytokines in ADSC-CM vary, depending on the culture conditions. Several studies demonstrated that ADSC-CM could be obtained in vitro under normal oxygen conditions (20–21%); however, other studies reported that under hypoxic conditions (2% O_2_), ADSC-CM is much richer in cytokines. It is possible that, under hypoxic conditions, ADSCs activate hypoxia-inducible factor 1α (HIF-1α), which can mediate various downstream signaling pathways [67], thereby stimulating ADSC proliferation and promoting the secretion of vascular endothelial growth factor (VEGF), hepatocyte growth factor (HGF), and basic fibroblast growth factor (bFGF) [29, 68]. Additionally, ADSC-CM obtained using three-dimensional culture, which better simulates the in vivo ADSC environment [69], contained more transforming growth factor-β1 (TGF-β1) and VEGF [44]. Thus, hypoxia, combined with three-dimensional culture, may provide optimal culturing conditions for obtaining ADSC-CM.

#### ADSC-CM and wound healing

Wound healing is a complex process, involving interactions between infiltrating cells, resident cells, and cytokines. The migration and proliferation of keratinocytes, endothelial cells, and fibroblasts and the deposition of ECM are considered the main biological processes involved in wound healing. Numerous studies showed that treatment with ADSC-CM may stimulate wound healing. ADSC-CM was found to promote the proliferation and migration of fibroblasts and accelerate the healing of ischemic wounds in adult rats [30]. Using an autologous plasma solution, which is abundant in growth factors, cytokines, and chemokines [70], Stessuk et al. [31] confirmed that platelet-rich plasma and ADSC-CM had significant effects on cell proliferation in vitro and contributed to tissue regeneration and repair. Zhao et al. [15] examined the cytokines in ADSC-CM that influenced skin fibroblast proliferation and migration and confirmed that ADSC-CM had high concentrations of epidermal growth factor (EGF), platelet-derived growth factor (PDGF)-AA, VEGF, and bFGF. Among these, bFGF was the main promoter of fibroblast proliferation and migration, whereas VEGF and PDGF-AA primarily promoted fibroblast migration. Compared with sole cytokine use, the combination of growth factors found in ADSC-CM more significantly promoted fibroblast proliferation and migration. Thus, the various factors present in ADSC-CM exert synergistic effects and jointly promote wound healing.

Moreover, depending on culture conditions, adipose stem cells show different effects on wound healing. Thus, compared with that obtained using two-dimensional culture, ADSC-CM from three-dimensional culture showed a superior stimulatory effect on the proliferation and migration of fibroblasts and keratinocytes and was more effective in promoting wound healing [32]. This was consistent with the finding that galectin-1 was only detected in ADSC-CM from three-dimensional culture, which was found to be an important factor for the migration of keratinocytes [32], as well as to the higher cytokine secretion [44]. Additionally, Lee et al. [29] studied the wound-healing effect of ADSC-CM obtained under hypoxic conditions, which was confirmed to contain higher amounts of secreted VEGF and bFGF. Importantly, wound area and depth were significantly reduced in mice treated with the hypoxic conditioned medium.

Besides ADSC-CM, adipose SVF-gel-conditioned medium (gel-CM) has been researched extensively in recent years. SVF-gel is a new adipose tissue graft; it contains natural ECM and ADSCs and is extracted by purely physical methods. Studies revealed that gel-CM contains higher concentrations of cytokines; it causes faster wound healing than CM obtained using adipose tissue and SVF cells [33, 34]. However, the specific reasons for the higher levels of cytokine secretion by SVF-gel require further investigation.

#### ADSC-CM and skin aging

Depending on its cause, skin aging can be classified into endogenous and exogenous aging. Endogenous aging is associated with the natural aging process of the human body, wherein fibroblasts are reduced in number and gradually lose their ability to multiply and synthesize collagen [71], which results in wrinkles, sagging skin, reduced skin elasticity, and collagen loss. Paracrine factors (EGF, bFGF, TGF-β, and VEGF) present in ADSC-CM promote the proliferation and migration of skin fibroblasts and increase the production of ECM and collagen, thereby improving the facial signs of aging, such as loss of skin elasticity [72].

For in vivo studies, ADSC-CM needs to penetrate the skin barrier. It is well known that the cuticle is the outermost layer of the epidermis and the main structure that resists external environmental damage and prevents water loss. It also prevents the penetration of various cosmetics and macromolecular drugs into the dermis. Hence, lasers and microneedles are often used in the clinic to create microchannels to increase drug absorption. ADSC-CM, combined with fractional carbon dioxide laser resurfacing treatment, improved skin aging by increasing skin elasticity, improving skin surface roughness, and reducing transepidermal water loss; moreover, ADSC-CM reduced pigmentation after laser treatment [35]. Furthermore, microneedles, combined with ADSC-CM treatment, reduced skin roughness, melanin content, and wrinkles and increased skin brightness, gloss, and elasticity [36]. Thus, cytokines present in the CM could enter the dermis through the generated micropores, thereby activating fibroblasts, stimulating collagen production and remodeling, and promoting skin regeneration.

During long-term exposure to the external environment, ultraviolet (UV) radiation is an important factor that causes skin aging, also known as exogenous aging or photoaging [73]. UV light upregulates the expression of matrix metalloproteinases (MMPs) and the secretion of proinflammatory cytokines by generating reactive oxygen species (ROS). UV also induces fibroblast damage in the dermis, thereby reducing ECM production [74]. It was also reported that the expression of type 1 procollagen was significantly upregulated and that of MMP-1 was significantly downregulated in a coculture of ADSC-CM and UV-irradiated human dermal fibroblasts (HDFs) compared with that in the control group [16]. Additionally, Guo et al. [17] explored the protective effect of ADSC-CM on fibroblasts against UVB irradiation. These researchers used HDFs at different degrees of senescence, designating the 5th, 15th, and 28th generations as young, intermediate, and senescent cells, respectively. These cells were pretreated with ADSC-CM and then irradiated with UV rays. The protective effect of ADSC-CM pretreatment was significantly reduced with HDF aging. Meanwhile, ADSC-CM upregulated the expression of type I and III collagen (Col1 and Col3, respectively) and elastin and downregulated the expression of *MMP1* and *MMP9* mRNA, with cells at the three degrees of senescence showing similar trends. Li et al. [37] further described the antiphotoaging mechanism of ADSC-CM using UVB-irradiated human keratinocytes and human skin fibroblasts. ADSC-CM reduced the production of MMP-1 and the secretion of IL-6 by downregulating the UVB-induced mitogen-activated protein kinase (MAPK) and TGF-β/Smad signaling pathways, thereby protecting both types of cells from UVB-induced photoaging.

Thus, as the main source of ECM proteins, which provide strength and toughness to the skin, fibroblasts play a vital role in both endogenous and exogenous skin aging. They may also provide a breakthrough in the study of the mechanism and treatment of skin aging. Analysis and application of specific conditioned medium components should be the focus of future research.

#### ADSC-CM and scars

Scars can be divided into pathological and physiological scars. Pathological scarring mainly refers to keloids and hypertrophic scars; inhibition of keloid formation by ADSC-CM has been reported. Wang et al. [38] suggested that the expression of tissue inhibitor of MMP-1 (TIMP1) and the deposition of Col1 in keloid tissue were significantly reduced after coculture of keloid tissue with ADSC-CM in vitro. Additionally, the number of CD31^+^ and CD34^+^ vessels was significantly reduced. Thus, ADSC-CM exerted an anti-scarring effect, by regulating collagen degradation and alleviating the abnormal deposition of collagen and the increase in keloid blood vessel density.

Hypertrophic scars are usually characterized by excessive deposition of ECM. Using a rabbit ear hypertrophic scar model, it has already been described that, after injecting ADSC-CM, the scar became flatter and thinner, while collagen fibers were arranged regularly and collagen deposition was reduced [39]. Li et al. [40] showed that ADSC-CM could reduce the expression of Col1, Col3, and β-smooth muscle actin (β-SMA) in vitro, thereby reducing collagen deposition and scar formation. These results were similar to those of an in vitro study performed by Chen et al. [41], who indicated that the proliferation and migration of hypertrophic scar fibroblasts were significantly suppressed by treatment with ADSC-CM and that the expression levels of ECM molecules decreased in these cells. Additionally, the treatment of hypertrophic scar fibroblasts with different concentrations (10%, 50%, and 100%) of ADSC-CM revealed that high concentrations of ADSC-CM could reduce the Col1/Col3 ratio and TIMP1 levels and upregulate MMP-1 expression [18]. Li et al. [40] further revealed that ADSC-CM has an anti-scarring effect by inhibiting the p38 MAPK signaling pathway, which plays an important role in hypertrophic scar fibrosis. Moreover, HGF in ADSC-CM plays a vital role in inhibiting the development of hypertrophic scar fibroblasts by regulating fibrosis factors and ECM remodeling [18]. Furthermore, the therapeutic effect of ADSC-CM against acne vulgaris scars was also described [37], almost all acne scars were healed in a rabbit ear acne scar model after ADSC-CM injection. The epidermis and stratum corneum became thinner, and the levels of tumor necrosis factor-α (TNF-α), IL-1α, and MMP-2 decreased in the ADSC-CM group. Thus, ADSC-CM reduces inflammation by inhibiting the production of inflammatory factors, thereby reducing scar formation [42].

Overall, ADSC-CM plays an indispensable role in reducing scar formation by promoting ECM decomposition and alleviating collagen deposition as well as by exerting anti-inflammatory and antifibrotic effects. It is speculated that the ability of ADSC-CM to reduce the formation of scar tissue is attributed to the cytokines present in the conditioned medium.

#### ADSC-CM and neuroprotection

In recent years, the use of ADSC-CM for the repair of nerve injury has also been reported. Peng et al. [43], using an in vitro model of glutamate excitotoxicity, confirmed that ADSC-CM exerted a neuronal protective effect. The release of lactate dehydrogenase (LDH) and the number of neuronal trypsin-positive cells were significantly reduced in the ADSC-CM treatment group; moreover, the level of apoptosis was lower than that in the glutamate-treated group. Additionally, ADSC-CM increased the number of CD31-positive microvessels and reduced that of microglial Iba1/TUNEL double-positive cells and the immunoreactivity of the glial fibrillary acidic protein (astrocytosis), thereby promoting the recovery of nerve tissue [44]. Moreover, ADSC-CM could reverse the glutamate-induced downregulation of a neural recovery marker, growth-associated protein 43 (GAP-43), and increase the levels of ATP, NAD, and NADH, thus rescuing the glutamate-induced neuronal energy depletion [43]. Cho et al. [44] examined the brain function and structure of rats on day 8 after the successful establishment of a rat stroke model and confirmed that continuous infusion of ADSC-CM for 7 days could significantly promote the rat brain function and structural recovery after stroke. Overall, it is speculated that the neuroprotective effect of ADSC-CM is attributed to the inhibition of apoptosis, reduction of neuronal energy depletion, promotion of cerebral angiogenesis, and reduction of astrocyte proliferation.

Neonatal hypoxic-ischemic encephalopathy has a relatively high morbidity and mortality in China; it is one of the main causes of acute neonatal death and chronic nervous system injury. Importantly, studies have reported the protective effect of ADSC-CM in a rat model of brain hypoxic-ischemic injury [19], the results proved that neurotrophic factors—insulin-like growth factor-1 (IGF-1) and brain-derived neurotrophic factor (BDNF), which are contained in ADSC-CM—played a critical role in the recovery from neuropathic injury and significantly reduced the impairment of long-term functional cognition and motor skills. Salgado et al. [75] analyzed the soluble factors secreted by ADSCs in various microenvironments and found that ADSCs could secrete growth factors closely related to neural regeneration, such as nerve growth factor (NGF), glial-derived neurotrophic factor (GDNF), and BDNF. These nerve regeneration-related growth factors may be the major contributors to the promotion of nerve injury repair by ADSC-CM.

#### ADSC-CM and the respiratory system

The respiratory system is another organ system in which ADSC-CM has shown promise. Acute respiratory distress syndrome (ARDS) is the major cause of death in clinically severe patients. Studies showed that paracrine factors present in ADSC-CM can effectively reduce the development of lung injury in patients with ARDS. ADSC-CM was found to inhibit histological changes in inflamed lungs and protein extravasation into bronchoalveolar lavage fluid, reducing the accumulation of the inflammatory mediators, TNF and IL-6, in a lipopolysaccharide-induced ARDS mouse model [45]. Additionally, pulmonary hypertension (PH) and pulmonary fibrosis (PF) are incurable diseases of the respiratory system; existing drug interventions have failed to improve clinical outcomes or reduce disease-related mortality. Rathinasabapathy et al. [46] proposed that ADSC-CM could improve monocrotaline-induced PH by increasing lung blood flow and inhibiting cardiac remodeling. ADSC-CM could also prevent the progression of PF in a bleomycin-induced model of lung fibrosis by reducing collagen deposition. ADSC-CM has great potential for the clinical improvement of PH and PF and may provide a new approach to their clinical treatment; however, the specific mechanism still needs to be further investigated.

### ADSC-Exo

#### Harvesting ADSC-Exo

Exosomes are extracellular membrane vesicles (diameter, 30–150 nm); they are released into the extracellular space through the fusion of polyvesicular bodies and the cytoplasmic membrane [76]. There are many harvesting methods for ADSC-Exo, including sucrose gradient centrifugation, based on exosomal size and density [14]; ultrafiltration, based on separation using membranes with different pore sizes [77]; immunoaffinity magnetic bead separation, based on exosomal surface marker expression [78]; and ExoQuick extraction, based on the polymer coprecipitation principle [79]. The most commonly used method is ultracentrifugation, based on exosome size [80]. When in vitro cultured ADSCs reach approximately 70 to 80% confluence, the cells are rinsed with phosphate-buffered saline (PBS) and cultured in fresh serum-free medium for 48 h; this is followed by the collection of the culture supernatant at 4 °C. The supernatant is centrifuged at 300×*g* for 10 min, 2000×*g* for 10 min, and 10,000×*g* for 30 min to remove cells and cell debris. The supernatant is then centrifuged at 100,000×*g* for 70 min; the pellet formed on the bottom of the centrifuge tube is resuspended in PBS, rinsed, and finally centrifuged at 100,000×*g* for 70 min to remove contaminating proteins and obtain exosomal particles (Fig. 1).

#### ADSC-Exo and myocardial protection

The rapid recovery of blood flow perfusion in the ischemic myocardium is an effective treatment for acute myocardial infarction (MI) [81]; however, ischemia/reperfusion may cause oxidative stress and an inflammatory response, leading to further damage to, and apoptosis of, myocardial cells [82]. Recently, several studies reported that exosomes from different stem cells can protect myocardial cells and enhance myocardial function [83, 84]. The therapeutic effect of ADSC-Exo against myocardial cell ischemia/reperfusion injury was also demonstrated [47]. ADSC-Exo were shown to be taken up by mouse cardiomyocytes and significantly inhibit H_2_O_2_-induced cardiomyocyte apoptosis, indicating that exosomes can protect mouse cardiomyocytes from oxidative stress-induced apoptosis. Additionally, Cui et al. [48] suggested that ADSC-Exo could significantly inhibit the expression of Wnt3a, β-catenin, and p-GSK-3b (Ser9) in vitro. The study also showed that the Wnt/β-catenin inhibitor XAV939 could partially abrogate the inhibition of cardiomyocyte apoptosis and the promotion of cardiomyocyte viability by ADSC-Exo, indicating that ADSC-Exo exert a protective effect on the myocardium by activating the Wnt/β-catenin signaling pathway. In a mouse ischemia/reperfusion injury model, the area of MI and serum CK-MB, LDH, and cTnI levels, as well as caspase-3 activation, were significantly decreased by ADSC-Exo [48].

Additionally, ADSC-Exo shows a significant protective effect against myocardial injury after MI [49]. Deng et al. [50] observed that ADSC-Exo reversed the MI-induced downregulation of sphingosine-1-phosphate (S1P), sphingosine kinase 1 (SphK1, also known as SK1), and sphingosine-1-phosphate receptor 1 (S1PR1), showing that S1P/SK1/S1PR1 signaling also participates in myocardial protection. Furthermore, in animal models, ADSC-Exo could partially alleviate the MI-induced decrease in cardiac function and cardiac structural changes. Moreover, ADSC-Exo reduced the MI-induced expression of the M1 macrophage markers IL-1, IL-6, TNF-α, and IFN-γ and promoted that of the M2 macrophage markers Arg1, Ym1, TGF-β1, and IL-10, suggesting that ADSC-Exo exert their anti-inflammatory effects by inhibiting M1 and promoting M2 polarization [50]. Further investigation of the protective effect of the active RNA present in ADSC-Exo against MI revealed that miR-146a-rich exosome groups were more effective in inhibiting AMI-induced apoptosis, inflammatory response, and fibrosis by suppressing early growth response factor 1 (EGR1) [20].

Overall, ADSC-Exo exert obvious protective effects on the myocardium by alleviating myocardial fibrosis and inhibiting apoptosis and inflammation, which may be related to the miRNA contained in the exosomes.

#### ADSC-Exo and neuroprotection

The effect of ADSC-Exo on neural regeneration was also studied. Bucan et al. [22] data demonstrated that ADSC-Exo could increase the axon length of dorsal root ganglion neurons, promote axonal growth, and regenerate damaged nerves. Additionally, ADSC-Exo could be easily internalized by Schwann cells and significantly promoted their proliferation and migration. Furthermore, ADSC-Exo-treated Schwann cells secreted more neurotrophic factors, such as NGF and BDNF [51]. Studies also demonstrated that nerve growth factors—BDNF, IGF-1, fibroblast growth factor-1 (FGF-1), and GDNF—were present in ADSC-Exo [22], which may account for the ability of ADSC-Exo to promote nerve regeneration.

As the main immune cells of the central nervous system, microglial cells can cause secondary nerve damage by generating internal stimulating factors, which act on surrounding cells after activation. ADSC-Exo pretreatment could inhibit the activation of microglial cells and reduce their cytotoxicity, which was associated with the inhibition of the NF-κB and MAPK pathways [52]. Additionally, Yang et al. [21] confirmed that ADSC-Exo could promote angiogenesis in vitro, whereas miR-181b-5p-enriched ADSC-Exo could enhance this effect. The underlying mechanism of the ADSC-Exo miR-181b-5p effect may involve the inhibition of the expression of TRPM7 and the improvement of the angiogenic function of brain microvascular endothelial cells, which provides a theoretical basis for stroke treatment.

ADSC-Exo also shows obvious protective effects in neurodegenerative diseases. Huntington’s disease is an autosomal dominant hereditary neurodegenerative disease caused by the aggregation of mutant Huntingtin (mHtt) [85]. Lee et al. [53] established an in vitro Huntington disease model and found that ADSC-Exo treatment could reduce the accumulation of mHtt aggregates, improve mitochondrial dysfunction, and reduce the rate of apoptosis. Additionally, ADSC-Exo could decrease the aggregation of mutant superoxide dismutase 1 (SOD-1) in G93A neurons, reduce abnormally expressed mitochondrial functional proteins, and restore the normal cell phenotype of amyotrophic lateral sclerosis (ALS), suggesting a therapeutic potential of ADSC-Exo in ALS [54]. Alzheimer’s disease is caused by the abnormal accumulation of amyloid-β peptide (Aβ) in the brain owing to an imbalance between Aβ production and clearance [55]. Neprilysin (NEP), a key degradation enzyme involved in Aβ hydrolysis and removal which is highly active in ADSCs-Exo, can be transferred to the neuroblastoma cell line N2a to reduce its intracellular Aβ level and reduce the accumulation of Aβ in the brain [55]. Thus, ADSC-Exo has therapeutic potential in nerve damage and neurodegenerative diseases.

#### ADSC-Exo and wound healing

Several studies showed the promising effects of ADSC-Exo in accelerating wound healing, which were mediated through the proliferation and migration of various cells. It is worth mentioning that exosomes could be internalized by fibroblasts, leading to an increase in the gene expression of N-cadherin, cyclin-D1, proliferating cell nuclear antigen (PCNA), Col1, and Col3 and the simultaneous promotion of cell proliferation and migration [56]. Interestingly, intravenous exosome administration was more effective in facilitating wound healing than local injections [56]. Zhang et al. [57] reported that exosomes stimulated the proliferation and migration of dermal fibroblasts, mainly by activating the PI3K/Akt signaling pathway. In addition to fibroblasts, ADSC-Exo also stimulates keratinocytes. Ma et al. [58] established a skin injury model by treating HaCaT cells with hydrogen peroxide and proposed that ADSC-Exo reduced apoptosis and promoted the migration of damaged HaCaT cells by activating Wnt/β-catenin signaling. Yang et al. [59] found that high expression levels of microRNA-21 in ADSC-Exo promoted wound healing by enhancing MMP-9 expression and inhibiting TIMP2 expression. Moreover, high miR-21 expression levels could downregulate TGF-β1 protein levels, thereby reducing the formation of scars in the wound. Additionally, the mouse skin wound-healing effect of exosomes, combined with hyaluronic acid (HA) treatment, was more obvious than that of exosomes alone [60]. Histological staining showed full-thickness skin regeneration and more significant angiogenesis in the ADSC-Exo + HA group. Furthermore, Li et al. [61] confirmed that ADSC-Exo, which overexpress Nrf2, could significantly stimulate the healing of foot wounds in diabetic rats by promoting the proliferation and angiogenesis of vascular endothelial progenitor cells and inhibiting the inflammatory protein expression and ROS production, which were associated with Nrf2-induced oxidative stress reduction and apoptosis during wound healing [61].

Overall, ADSC-Exo can promote wound healing through a variety of mechanisms involving cell proliferation and migration, including the activation/inhibition of the PI3K/Akt, Wnt/β-catenin, and other signaling pathways. The specific mechanism underlying the ability of ADSC-Exo to promote wound healing still needs further research; however, it is undeniable that the significance of ADSC-Exo effects has presented a new opportunity to study wound healing in recent years.

#### ADSC-Exo and atopic dermatitis

Atopic dermatitis, also known as atopic eczema, is characterized by increased serum IgE levels and increased eosinophil counts in the peripheral blood; it is often manifested as dry skin, eczema-like rash, and severe itching. ADSC-Exo can suppress the occurrence and development of atopic dermatitis by regulating the expression of inflammatory cytokines [62]. ADSC-Exo was applied to a mouse model of atopic dermatitis, which was induced by Biostir®-AD cream containing a dust mite antigen. Compared with the effect of oral prednisone tablets, both intravenous and subcutaneous injections of ADSC-Exo three times a week for 4 weeks significantly reduced the symptoms of atopic dermatitis in a dose-dependent manner. Mechanistically, ADSC-Exo injection significantly reduced, in a dose-dependent manner, the number of inflammatory cells (such as CD86^+^ and CD206^+^ cells), the serum IgE-mediated eosinophil counts in skin lesions, and the levels of *IL-4*, *IL-23*, *IL-31*, and *TNF-α* mRNA.

### ATE

#### Harvesting ATE

ATE is a cell-free fluid, which is abundant in cytokines and can be rapidly extracted from adipose tissue by purely physical methods [23, 24]. In brief, the obtained lipoaspirate is left in ice water, and the liquid portion is discarded. Then, the collected adipose tissue layer is washed with PBS and centrifuged to remove remaining blood cells and other components. The collected adipose tissue is mechanically emulsified and then centrifuged and filtered, either directly or after being frozen and thawed. The resultant liquid is the ATE (Fig. 1).

#### ATE and wound healing

The wound healing process is divided into the following stages: hemostasis, inflammation, proliferation, and remodeling. During the inflammation phase, neutrophils and monocytes are activated and infiltrate the wound area; after the conversion of monocytes into macrophages in the tissue, some growth factors and cytokines are produced to promote wound repair. During the proliferation and remodeling phases, the migration and proliferation of fibroblasts and the synthesis of ECM contribute to skin wound repair. Normal angiogenesis is particularly important for wound closure; the absence of blood vessels can lead to chronic wound formation [76, 86]. ATE can induce neovascularization, thus promoting wound healing [24, 25]. An in vitro study by Lopez et al. [28] showed that ATE contained high concentrations of KGF but low concentrations of bFGF, VEGF, EGF, IGF-1, IL-6, PDGF-B, TGF-β, and TNF-α, which may explain why ATE promotes keratinocyte proliferation and migration, adipose stem cell and fibroblast migration, and endothelial cell growth. Furthermore, Na et al. [63] indicated that ATE could promote skin wound healing in mice and the proliferation and migration of in vitro cultured skin fibroblasts in a dose-dependent manner. It is speculated that the extracellular vesicles and/or growth factors present in ATE stimulate tissue repair by promoting cell proliferation and migration, as well as angiogenesis, thereby accelerating wound healing.

#### ATE and skin aging

ATE also exerts an anti-aging skin effect by reducing UV ray-induced ROS production and promoting collagen synthesis. Deng et al. [64] showed that ATE could promote the proliferation of dermal fibroblasts, thereby increasing ECM synthesis and reducing UVB-induced intracellular ROS accumulation through the upregulation of the expression of the antioxidant enzyme glutathione peroxidase (Gpx-1), ultimately exerting antiphotoaging effects. Additionally, Zheng et al. [26] observed that the expression of MMP-1 and MMP-3 decreased and that of TIMP1 and TIMP3 increased in nude mice treated with ATE, indicating that ATE could promote ECM production, increase the thickness of the nude mouse dermis, and inhibit collagen degradation.

#### ATE and ischemic disease

Persistent tissue ischemia eventually leads to tissue necrosis; therefore, early blood supply restoration is essential for tissue repair. The data obtained in a mouse hindlimb ischemia model, established by Yu et al. [23], suggested that hindlimb ischemia in mice was significantly improved by both high and low concentrations of ATE compared with that in the PBS-treated group. Immunohistochemistry revealed that there were more CD31-positive capillaries in the ATE treatment group. Additionally, Cai et al. [27] established a rat skin flap model and found that ATE promotes flap angiogenesis and increases its survival rate. Thus, ATE has the ability to promote angiogenesis, which is supposedly attributed to the presence of angiogenesis-related growth factors in ATE.

#### ATE and epilepsy

Epilepsy is a common disease of the nervous system; it is characterized by intermittent abnormal electrical activity, cell loss or neurodegeneration, and brain inflammation. Jeon et al. [65] found that ATE had an antiepileptic effect. The number of spontaneous recurrent episodes of epilepsy and epileptic behavior in a pilocarpine-induced mouse epilepsy model could be reduced by ATE. Additionally, long-term use of ATE could inhibit or even prevent the occurrence of epilepsy. Microarray experiments revealed that this long-term effect appeared to correct abnormal inflammatory neuronal circuits in the epileptic brain to normal neuronal circuits by reducing inflammatory responses and neuronal excitability [65].

#### Storage and administration of cell-free derivatives

Compared with adipose stem cells, ADSC-CM, ADSC-Exo, and ATE have a simpler storage method and do not need to be stored in a toxic cell cryopreservation solution, which avoids cell damage. However, the storage of the three cell-free derivatives is also different, lacking a unified storage method. For example, ATE is generally stored at − 20 °C, although some authors have indicated its storage at − 40 °C [24]; extracted ADSC-Exo is suspended in PBS or PBS containing Exo and stored at − 80 °C, while ADSC-CM is stored at − 20 °C or − 80 °C. In addition, as far as the method of administration is concerned, these three cell-free derivatives are usually mainly applied topically, and some are given by subcutaneous injection or intravenous injection. However, regardless of the application method, no adverse reactions seem to be found.

#### Comparison of three cell-free derivatives

Compared with stem cell therapy, the three cell-free derivatives contain no cells. ADSC-CM mainly contains cytokines, exovesicles, and exosomes secreted by ADSC. Exo mainly carries proteins secreted by ADSC and active molecules such as mRNAs, microRNAs, lncRNA, DNA, etc., which are internalized by receptor cells through receptor-ligand binding or fusion with plasma membrane and endocytosis mechanism to exchange protein and gene information [87], thereby promoting tissue damage repair. ATE is easier to obtain and is richer in cytokines and extracellular vesicles. Therefore, it can be assumed that these derivatives are non-immunogenic, which can promote their use in the same species or in different species of animal. Second, the use of cell-free derivatives overcomes challenges such as low survival rate of implanted cells or the potential risk of tumorigenesis. Further, as mentioned above, cell-free derivatives do not require the use of cell cryopreservation solution, are easy to store and transport, and have great advantages in clinical application. However, there are some problems with the application of these cell-free derivatives. For example, because cell-free derivatives do not contain cells, compared with stem cell therapy, the efficacy of cell-free derivatives is not as good, and multiple injections are needed to achieve better therapeutic effects. Moreover, the main functional molecules in ADSC-Exo are still unclear, and there is currently no unified dosage and method of administration or storage scheme for cell-free derivatives; thus, more systematic and extensive research is needed to solve these problems.

### Limitations

Significant progress has been achieved in the treatment of many diseases using cell-free derivatives, which have become an ideal solution for cell-free therapy in the field of regenerative medicine; however, some questions remain to be addressed.

First, ADSC-Exo are extracellular vesicles that carry a variety of cargo—proteins, RNA, and DNA [14]; however, the specific functions and applications of the cargo have not been fully studied.

Second, Hu et al. [56] found that the intravenous injection of exosomes showed a better effect on wound healing than local injection, whereas Cho et al. [62] reported that both intravenous and subcutaneous injections significantly alleviated the symptoms of atopic dermatitis in a dose-dependent manner. Hence, it is necessary to conduct further research to establish a uniform exosome administration procedure.

Third, because cell-free derivatives do not contain stem cells, multiple injections are required to maintain the desired effects, which may limit the application of adipose cell-free derivatives to a certain extent.

Lastly, as most existing studies are based on animal research, there is a lack of clinical research in humans, especially regarding the role of ATEs. Therefore, clinical studies are still needed for further investigation in the future.

## Conclusions

In summary, the use of adipose cell-free derivatives overcomes the limitations of the clinical applications of ADSCs and shows a number of advantages. These adipose derivatives are cell-free and easy to carry, transport, and store; they display low immunogenicity and no potential tumorigenicity and can thus be used for allogeneic transplantation, which makes adipose cell-free derivatives better candidates for commercial promotion. Local transplantation can promote cell proliferation, migration, and angiogenesis; suppress cell apoptosis and inflammation; and reduce oxidative stress and immune regulation. Therefore, cell-free derivatives have broad clinical application prospects and are widely used in anti-aging, wound healing, scar repair, and nerve regeneration research. However, the preparation of ADSC-CM and ADSC-Exo currently requires cell isolation and in vitro culture, which may result in biological contamination. Additionally, the preparation of ADSC-Exo involves cumbersome and time-consuming steps and needs further improvement.

The ATE rich in cytokines and extracellular vesicles obtained by pure physical methods does not require cell separation and in vitro cultivation and can be quickly prepared in the operating room or simple laboratory, significantly reducing the risk of biological contamination. As such, compared with ADSC-CM and ADSC-Exo, the use of ATE may be easier to promote in clinical application. In addition, studies have shown that compared to conditioned medium derived from adipose tissue and stromal vascular segment cells, gel-CM does not require xenogenic collagenase digestion and in vitro expansion, and its effect of promoting wound healing is better [33, 34], indicating that ADSC may secrete more high concentrations of cytokines under the protection of natural extracellular matrix. In addition, gel-CM and ATE may also contain high concentrations of exosomes, which may be a new source of ADSC-Exo, and is expected to be widely used in tissue repair and regenerative medicine.

## Data Availability

Data sharing is not applicable to this article as no datasets were generated or analyzed during the current study.

## Abbreviations

Aβ: Amyloid-β peptide
ADSC: Adipose-derived stem cell
ADSC-CM: Adipose-derived stem cell-conditioned medium
ADSC-Exo: Adipose-derived stem cell exosomes
ALS: Amyotrophic lateral sclerosis
ARDS: Acute respiratory distress syndrome
ATE: Adipose tissue extract
BDNF: Brain-derived neurotrophic factor
bFGF: Basic fibroblast growth factor
Col1: Type I collagen
Col3: Type III collagen
ECM: Extracellular matrix
EGF: Epidermal growth factor
EGR1: Early growth response factor 1
Ex-4: Exendin-4
FGF-1: Fibroblast growth factor-1
MSC: Mesenchymal stem cells
GAP-43: Growth-associated protein 43
GDNF: Glial-derived neurotrophic factor
gel-CM: Fat gel-conditioned medium
Gpx-1: Glutathione peroxidase
HA: Hyaluronic acid
HDF: Human dermal fibroblast
HGF: Hepatocyte growth factor
HIF-1α: Hypoxia-inducible factor 1α
IGF-1: Insulin-like growth factor-1
LDH: Lactate dehydrogenase
MAPK: Mitogen-activated protein kinase
MI: Myocardial infarction
MMP: Matrix metalloproteinase
NEP: Neprilysin
NGF: Nerve growth factor
PBS: Phosphate-buffered saline
PCNA: Proliferating cell nuclear antigen
PDGF: Platelet-derived growth factor
PF: Pulmonary fibrosis
PH: Pulmonary hypertension
ROS: Reactive oxygen species
SOD-1: Superoxide dismutase 1
S1P: Sphingosine-1-phosphate
S1PR1: Sphingosine-1-phosphate receptor 1
SphK1 or SK1: Sphingosine kinase 1
β-SMA: β-Smooth muscle actin
SVF: Stromal vascular fraction
TGF-β: Transforming growth factor-β
TIMP: Tissue inhibitor of metalloproteinase
TNF-α: Tumor necrosis factor-α
VEGF: Vascular endothelial growth factor
UV: Ultraviolet
